# ClearSpeechTogether: a Rater Blinded, Single, Controlled Feasibility Study of Speech Intervention for People with Progressive Ataxia

**DOI:** 10.1007/s12311-022-01462-9

**Published:** 2022-08-24

**Authors:** Anja Lowit, Jessica Cox, Melissa Loucas, Jennifer Grassly, Aisling Egan, Frits van Brenk, Marios Hadjivassiliou

**Affiliations:** 1 School of Psychological Sciences and Health, Strathclyde University, 40 George St, Glasgow, G1 1QE Scotland; 2 School of Psychology and Clinical Language Sciences, Earley Gate, University of Reading, RG6 6AL Reading, England; 3 University College London, Chandler House, 2 Wakefield Street, London, WC1N 1PF England; 4 Department of Languages, Literature and Communication, Utrecht University, Trans 10, 3512 JK Utrecht, Netherlands; 5 Sheffield Teaching Hospitals NHS Trust, Royal Hallamshire Hospital, Glossop Rd, Sheffield, S10 2JF England

**Keywords:** Progressive ataxia, Dysarthria, Speech therapy, Group intervention, Intelligibility, Communication participation

## Abstract

**Background:**

Progressive ataxias frequently lead to speech disorders and consequently impact on communication participation and psychosocial wellbeing. Whilst recent studies demonstrate the potential for improvements in these areas, these treatments generally require intensive input which can reduce acceptability of the approach.

A new model of care—ClearSpeechTogether—is proposed which maximises treatment intensity whilst minimising demands on clinician. This study aimed to establish feasibility and accessibility of this approach and at the same time determine the potential benefits and adverse effects on people with progressive ataxias.

**Method:**

This feasibility study targeted people with progressive ataxia and mild-moderate speech and gross motor impairment. ClearSpeechTogether consisted of four individual sessions over 2 weeks followed by 20 patient-led group sessions over 4 weeks. All sessions were provided online. Quantitative and qualitative data were collected for evaluation.

**Results:**

Nine participants completed treatment. Feasibility and acceptability were high and no adverse effects were reported. Statistical tests found significantly reduced vocal strain, improved reading intelligibility and increased participation and confidence. Participant interviews highlighted the value of group support internalisation of speech strategies and psycho-social wellbeing.

**Discussion:**

ClearSpeechTogether presented a feasible, acceptable intervention for a small cohort of people with progressive ataxia. It matched or exceeded the outcomes previously reported following individual therapy. Particularly notable was the fact that this could be achieved through patient led practice without the presence of a clinician. Pending confirmation of our results by larger, controlled trials, ClearSpeechTogether could represent an effective approach to manage speech problems in ataxia.

## Introduction

Progressive ataxia is the term used to describe a number of different diseases that primarily affect the cerebellum resulting in loss of coordination, limb clumsiness, gait instability, falls, slurred speech and sometimes visual problems. The term tends to be used in the context of those ataxias that are not due to a structural pathology (e.g., tumour, stroke, multiple sclerosis, trauma). As the majority of ataxias are not treatable, patients accumulate significant disability over time, sometimes becoming wheel-chair dependant with reduced lifespan. The causes of progressive ataxias can be broadly divided into genetic, acquired (non-degenerative) and degenerative. The commonest inherited ataxia is Friedreich’s ataxia (FRDA). The prevalence of other genetic ataxias has considerable geographical variation. Non-degenerative acquired ataxias include immune ataxias (e.g., gluten ataxia, paraneoplastic cerebellar degeneration, post infectious cerebellitis), and the most notable example of a degenerative ataxia is cerebellar variant of multiple system atrophy (MSA-C). Depending on the aetiology, ataxias can progress rapidly (e.g., immune ataxias and MSA-C) or slowly over many years (e.g., genetic ataxias). Depending on aetiology some ataxias can be more commonly associated with dysarthria (e.g., MSA-C) whilst others can be more commonly associated with gait instability (e.g., immune ataxias).

As the disease progresses ataxia can lead to speech problems, presenting as ataxic dysarthria. The nature and onset of disease varies across and even within ataxia types. For example, Friedreich’s ataxia (FRDA) has been described as falling into three different categories of progression and symptomatology [1], and the same is true for dominantly inherited spino-cerebellar ataxias (SCA) [2, 3]. Despite these individual differences, ataxic dysarthria can generally be characterised by symptoms impacting on all speech sub-systems, i.e., respiration, laryngeal function, articulation and resonance [4–16], leading to reduced speech intelligibility and communication breakdown. This is likely to have further consequences on an individual’s quality of life. Studies on communication impact of dysarthria due to other neurological conditions such as Parkinson’s disease or following stroke suggest that speakers experience poor mental health, negative self-image and withdrawal from communication and thereby social contacts [17–20]. No such studies have been published for people with ataxia, however, more than a third of respondents to a recent survey by Ataxia UK identified speech problems as one of the three most troublesome symptoms of their disease [21].

Whilst our understanding of the nature of the communication problems experienced by people with progressive ataxia has improved significantly over time [1, 5, 7, 9, 12–15], a 2017 Cochrane Review on treatment efficacy for these syndromes concluded that insufficient and low quality evidence was available on the effectiveness of speech interventions to support this population [22].

Since the publication of the Cochrane review, further studies relating to speech treatment in progressive ataxia syndromes have been published. Together these studies have highlighted a range of potential communication benefits across all areas of the International Classification of Functioning and Disability (ICF) model, i.e., impairment (e.g. breath support, voice quality, loudness [23–28]), activity (intelligibility and naturalness, [24, 27, 29, 30]) and participation and communication confidence [27, 28, 31]. There is thus mounting evidence that speech intervention can have benefits both for speech and wider communication impact in people with progressive ataxias. However, most of the interventions have required relatively intensive input from clinicians, usually provided in individual patient settings. This is labour intensive and costly on the part of the health provider and can increase wait times for other patients.

An alternative model of care that addresses the demands on clinician’s time is group therapy. A recent systematic review on the benefits of this model in acquired dysarthria [32] found that it may increase treatment intensity and be potentially more cost-effective. The authors also highlight the increased opportunities for socialization, support and integration of more client-driven goals into the activities, and how practice in more naturalistic contexts as well as the social aspects of group intervention can facilitate better generalization of treatment targets and potentially also motor learning. More specifically, researchers have found that well-structured group therapy can provide similar quantitative benefits to individual therapy in primary intervention for speech deficits, as reflected by significant improvements in measures such as vocal intensity [33–37], maximum phonation time (MPT) [34, 35, 37, 38] and intelligibility [39, 40]. In addition, studies have demonstrated that group therapy can be effective in maintaining the gains resulting from intensive individual therapy [35, 41]. One aspect that has been highlighted as unique to group therapy is the social support patients provide to each other. This is reported to improve confidence and self-esteem [42–45]. In addition, participants may feel like they can contribute and participate more and tackle speech goals relevant to them in a more naturalistic settings [42, 46]. Such outcomes are particularly important in addressing the psychosocial impact reported in speakers with dysarthria such as loneliness and social isolation [17, 18, 20], which has been particularly exacerbated by the Covid-19 pandemic and the associated lockdown measures.

Whilst a number of studies suggest group intervention can lead to similar speech outcomes as individual therapy, this might come at a cost as studies have shown that higher dosage achieves better outcomes [47, 48], thus reducing the cost comparison between the two care models. To address this issue, we developed a novel treatment model—ClearSpeechTogether—that maximises treatment intensity whilst minimising clinician time. ClearSpeechTogether is a mixed individual—group therapy design. Its novelty lies in the fact that group sessions are facilitated by the patients themselves rather than trained clinicians, thus reducing pressure on health services whilst maximising opportunities to internalize speech strategies for patients in a supportive, naturalistic environment.

This study aimed to establish the basis for future larger investigations by piloting the effects of ClearSpeechTogether on people with progressive ataxia and communication difficulties, and to establish the feasibility and acceptability of the approach. Our research questions were as follows:

- RQ1: What is the feasibility of ClearSpeechTogether in a population of people with progressive
  ataxia?
  Outcome measures: recruitment, attrition, adherence, need for additional individual sessions
  RQ2: What is the acceptability of the approach to participants?
  Outcome measures: fatigue measures, qualitative participant feedback on delivery format.
  RQ3: What are the potential communication and psychosocial benefits, and adverse effects of the approach?

Outcome measures: maximum phonation time, voice quality, intelligibility, sentence production consistency, communication participation, communication confidence, qualitative participant feedback, clinician observations.

The study is reported according to CONSORT 2010 statement: extension to randomised pilot and feasibility trials [49].

## Materials and Methods

### Trial Design

This 12-month study was a rater-blinded, single cohort design of patients with dysarthria due to progressive ataxia, using a single study arm—ClearSpeechTogether. Participants acted as their own controls by implementing a 2-week no treatment phase. No adjustments were made to the methodology following registration of this study in the ISRCTN clinical trial database [50].

### Sample Size

The study was intended to function as a pilot study to establish suitability of the intervention approach for a larger RCT. For this purpose, it was decided to run two groups of five participants each, aiming for a total of ten recruits for the study.

### Participants

Eligibility criteria for the study included a confirmed diagnosis of progressive ataxia, the presence of mild to moderate predominantly ataxic dysarthria, the absence of a functional voice disorder other than can be expected as part of the ataxia, the absence of visual, hearing or cognitive impairments that would have impact on participation in the assessment or treatment regime, age above 16 years, and availability and ability to use the technology necessary to complete assessment and treatment sessions online via *Zoom.*

Advertising took place via the funder website and social media campaigns. In addition, people with ataxia who had requested to stay informed about upcoming trials from a previous study were contacted directly via email. All participants self-selected and were provided with study information by email after contacting the research team for more information about the study. For those still interested, suitability to participate was established during a Zoom call with the first author, during which consent was also taken for those recruited to the study.

### Study Design

Patient involvement in this study lasted for 16 weeks. This included a 2-week pre-therapy assessment period, 6 weeks of intervention and a further 8-week assessment period. Given the distance of study participants’ homes to the investigators and to each other, and the fact that the UK was undergoing various COVID-19 related lockdown measures at the time of the study, all assessments and individual and group therapy sessions were delivered remotely via Zoom. The feasibility of telehealth provision in this population had been established in our previous study using Skype [25].

Assessments required participants to record themselves at home. For this purpose, they were provided with information on how to use freely available recording software Audacity^R^ (version 3.0.3). Two participants had iPads and used the inbuilt voice recorder instead. Each participant was provided with a headset microphone to ensure stable mouth-to-microphone distance and a low-cost speech intensity meter (Cadrim Digital Sound Level Meter). They were sent a OneDrive link to securely upload their recordings after each assessment session. Backup recordings were made using Zoom cloud recordings with participants’ consent.

### Assessment Tasks

The study included multiple baseline assessments (sessions 1 & 2, administered 2 weeks apart prior to treatment), and two post-therapy assessments, one within 1 week of completing treatment, and another 8 weeks post-treatment (sessions 3 & 4). Assessments were conducted by the first author who was not involved in the treatment of participants.

In line with the ICF model, we assessed participant’s communication at impairment, activity and participation level. In addition, we collected information on fatigue and their medical history, as summarised in Table 1.

**Table 1:** Assessment summary

Task	Session	ICF level	Measure
Demographic and medical information	1		NA
Fatigue Impact Scale	1 and 3		Total score
Maximum phonation time	1–4	Impairment	Maximum duration in sec Perceptual voice quality
Voice quality	1–4	Impairment	GRBAS score
Reading passage	1–4	Activity	Intelligibility (DME)
Monologue	1–4	Activity	Intelligibility (percentage scale)
Sentence repetition	1–4	Activity	Utterance to utterance variability (UUV)
Communication Participation Item Bank	1 and 3	Participation	Total score (10 items, 0–3 scale)
Communication Confidence	1 and 3	Participation	Total score (1–10 scale)
Interview	1 and 3	Participation	Content Analysis

Fatigue was captured with the overall score of the Fatigue Impact Scale (FIS [51]). For speech, two repetitions of maximum phonation time were collected unless the participant clearly performed within the normal range with a duration of around 20 s or more on their first attempt. Where two attempts were collected, the better of the two was used for subsequent analysis. Connected speech samples were captured by a reading task and a spoken monologue. The reading passage comprised the first paragraph of the Caterpillar passage [52], which resulted in 20 to 30 s samples, and for the monologue participants were asked to talk about a topic of their choice, such as a holiday, a recent memorable event or a hobby for about one minute. In addition, we recorded ten repetitions of the sentence “Tony knew you were lying in bed” to measure the consistency of sentence production (utterance to utterance variability, UUV [53]). This measure had previously shown some promise of being sensitive to intelligibility levels and could thus potentially quantify any post-treatment improvements in this parameter.

During the post-treatment assessment sessions, all participants were explicitly requested to apply the speech strategies developed during the intervention phase.

Finally, we captured participation by asking participants to complete the Communication Participation Item Bank (CPIB [54]) and to score their level of communication confidence on a 10 point scale.

### Analysis

The primary speech outcomes measures were duration in the MPT, and intelligibility of connected speech (reading and monologue samples). Secondary outcomes included consistency of sentence production, measures of communication participation and confidence, fatigue ratings and patient perceptions. All examiners were blinded to the time point of the samples they analysed.

#### Vowel Prolongation

Vowel prolongation was analysed in terms of MPT and voice quality. Oscillographic and wide-band spectrogram data viewed in Praat ([44], version 6.0.43) were used for duration measures. In addition, four experienced speech and language therapists (SLTs) used the GRBAS [55] to provide perceptual evaluations of voice quality. This tool provides scores for Grade (G—overall severity), roughness (R), breathiness (B), asthenia (A—weak voice) and strain (S).

#### Connected Speech

Intelligibility in the reading and monologue tasks were rated by four experienced SLTs. Due to the repetitive nature of the reading material, listeners scored the samples using the Direct Magnitude Estimation (DME) method [56] using the first recording of the reading sample (session 1) as the standard. For the evaluation of the monologue, listeners scored the samples on a percentage scale. The samples comprised of around 30-s continuous speech without interruptions from the examiner or extraneous noise. As for reading, all samples were presented in randomised order of assessment but grouped by speaker.

#### Communication Participation

We conducted semi-structured interviews in sessions 1, 3 and 4 to establish the form, severity and impact of speech problems experienced by participants, and how these were affected by the intervention. We also asked them to complete the short form of the Communication Participation Item Bank (CPIB) [54] on these occasions, and to provide a single score on a scale of 1–10 of their confidence when communicating with people outside their immediate social circle.

#### Acceptability of the Approach

The participant interviews also focused on the content and presentation of the treatment, discussing areas such as appropriateness of the exercises, treatment intensity, group dynamics, online nature of presentation and balance of individual versus group input.

### Inter-rater Reliability and Statistical Analysis

To assess inter-rater reliability, we conducted an independent analysis of four participants for the various measures performed. Agreement was excellent with an intraclass correlation coefficient (ICC) of 0.999 for the MPT task. In addition, agreement between the four expert listeners for the perceptual analysis of the data was good with an ICC of 0.804 for reading intelligibility, and 0.884 for the monologue intelligibility, and 0.808 for voice quality.

Due to the small sample size and variability of speaker presentation non-parametric statistics were applied to avoid overinterpretation of results. The Friedman Test was performed to assess changes across time, using the Wilcoxon-signed rank test for the post hoc analyses. We chose not to employ Bonferroni corrections given the sample size and highly exploratory nature of the investigation, but considered these factors in the interpretation of the results. For correlational analyses we employed Spearman’s Rho. Listener agreement was calculated with the Intraclass correlation coefficient.

### Treatment Schedule

Treatment was administered over a period of 6 weeks. This included an initial 2 weeks of individual therapy with two sessions of 45–60 min per week (4 individual sessions) and twice daily homework tasks. This was followed by 4 weeks of intensive peer supported group practice, consisting of daily 1 h virtual meetings with the group (20 group sessions). The group phase was supported by a weekly meeting with the SLT. There was the option to provide further individual input for participants if the clinician determined that they were not using the speech strategies effectively or showed adverse reactions. A non-specialist volunteer was present during the non SLT-led group sessions to support the participants with any technical issues. The SLT-led sessions were administered by two expert clinicians who were highly experienced in treating patients with ataxia.

### Treatment Focus

In line with previous trials for people with progressive ataxia [25, 27, 29, 57], two global speech strategies were focused on in this study – LOUD and CLEAR. Principles of the Lee Silverman Voice Treatment (LSVT LOUD®) programme were adopted in terms of the focus on voice, however, unlike in Parkinson’s disease, volume is not necessarily in issue for people with ataxia. Even though we maintained the cue “LOUD” for participants, it represented effective voice use and clinicians also ensured that voice quality was not strained or effortful, and produced at the appropriate pitch. “CLEAR” speech production aimed to maximise intelligibility for a communication partner by encouraging participants to over-articulate. The individual sessions were used to introduce participants to the two therapy concepts and to establish their use at least at single word level. The group phase then involved participants working through a handbook of graded exercises in line with the LSVT LOUD® programme. These briefly involved further practice at the single word level and then moved quickly on to phrases, sentences and increasingly complex reading and free speech exercises by the end of week 4. Participants also practised ten daily phrases during the sessions, and completed prolonged vowel exercises as a warm up before the group meeting, to maximise time during the session for targeted speech activities.

Participants were provided with materials to practise, but also increasingly asked to prepare their own materials to build independence during the post-therapy phase for continued practice. The SLT met with the group at the end of each week to monitor each participant’s progress, suggest adjustments as necessary and to explain the upcoming tasks for the following week. The weekend was available for participants to prepare materials as necessary.

Participants were invited to reflect and comment on each other’s performance in a constructive way. This was intended to provide support but also developed participants’ ability to monitormselves and others. All exercises were designed to be executed in turns, ensuring active involvement of all participants throughout the session. In addition, participants rotated as “session chair”, which involved time management and ensuring all exercises were attempted. They were also responsible for contacting the research team if they were unclear about any exercise or if any other problems arose.

Speech exercises were designed to last 20 to 30 min though sessions tended to last 45 to 60 min depending on how much social chat was included at the start and end of the meeting..

## Results

### Recruitment

The recruitment period lasted 3 weeks. We had an unprecedented level of interest in the study, with more than 50 people with ataxia interested in participating. Eligible candidates were contacted in the order they approached the research team until all ten places were filled. One person had to be rejected due to a co-morbidity presenting during this process.

### Baseline Data

Twelve patients with progressive ataxia were recruited. Their details, including medical history and dysarthria features, are summarised in Table 2. As can be seen, ataxia diagnosis varied considerably across participants. As a result of COVID-19 lockdown measures and the resulting impact health services, no up to date neurological examinations were conducted as part of this study. Instead, we relied on patient reports of their medical history and used a broad grading of their motor disability as stage 0 = no gait difficulties, stage 1 = disease onset, as defined by onset of gait difficulties; stage 2 = loss of independent gait; stage 3 = confinement to wheelchair; stage 4 = death [58] which was deemed sufficient for the purposes of this pilot study which focused entirely on their speech performance. Speech severity was derived from the intelligibility ratings of the monologues. The majority of our participants were rated as showing mild-to-moderate gross motor impairment (stages 1 & 2, Table 2). In addition, most had a mild to mild-moderate level of speech impairment, with only two located at the lower moderate-to-severe end of the spectrum (AD8 & AD9, Table 2). No specific information was gathered from participants regarding their educational level, and no formal cognitive assessment was conducted. However, all participants had held employment suggesting education to at least high school level and none showed any notable signs of cognitive decline that could have impacted on their participation. All were highly familiar with Zoom, having used it throughout the previous COVID-19-related lockdown period.

**Table 2:** Participant characteristics

Participant	Age	Gender	Diagnosis	Years since diagnosis	Motor impairment	Intelligibility deficit (% scale)
AD1	56	M	SCA28	2	1	74
AD2	70	M	Idiopathic cerebellar ataxia	6.5	1	71
AD3	NA	NA	NA	NA	NA	NA
AD4	56	M	Idiopathic cerebellar ataxia	1	2	78
AD5	64	F	CANVAS	9	2	75
AD6	66	M	Idiopathic cerebellar ataxia	1	1	79
AD7	NA	NA	NA	NA	NA	NA
AD8	46	M	SCA3	17	3	45
AD9	56	M	Presumed autoimmune ataxia	3	2	51
AD10	57	F	Friedreich’s ataxia	10	2	66
AD11	66	F	SCA6	5	2	69
AD12	NA	NA	NA	NA	NA	NA

### Adherence

Of the twelve patients recruited, eleven commenced and nine completed treatment. Retention was not impacted by the intervention approach, but rather significant personal circumstances. We were able to replace the first participant as no treatment had been started (AD3 with AD6). In the second case (AD7) the participant had already completed the individual therapy phase and no replacement was possible. In order to maintain the same group size, we admitted a further participant, AD12, to join the group sessions in place of AD7 on day 2 of the group phase after providing a brief introduction to the treatment strategies. His data are not reported in this study.

Adherence to treatment was generally good, two thirds of participants attended all sessions, the remaining three (AD5, AD8 and AD11) missed a maximum of 4 group sessions.

### Numbers Analysed

Of the nine participants completing the full programme, eight were included in the analysis. AD11 was diagnosed with a medical condition that could have affected pre-treatment assessment after the individual therapy had been completed. In addition, poor recording quality necessitated the removal of AD8’s final assessment data with the exception of his MPT performance which could be reliably extracted.

### Outcomes

#### Fatigue

Global fatigue scores from the FIS ranged from 4 to 10 (with ten being normal) at first assessment, with a mean of 6.3 (SD = 2.1). Comparison with scores at assessment 3 (mean = 7.6 (SD = 1.9)) suggested no significant change (*p* = 0.680).

#### Maximum Phonation Time

Maximum phonation time demonstrated no post-treatment effects (*p* = 0.366, *χ*^2^ = 3.17, *df* = 3). However, it should be considered that most participants’ performance fell within the normal range [59, 60], achieving MPTs of up to 30 s. Both participants who performed below the norm did in fact show a small increase in performance post-therapy, moving from 6 to 13 s (AD5) and 9 to 13 s (AD10) between assessment 1 and assessment 4.

#### Voice Quality

Most dimensions on the GRBAS showed no significant changes across the assessment points. However, strain was significantly reduced between between A2 and the immediate and 8 week follow up assessments (Friedman test: *p* = 0.010, *χ*^2^ = 11.43, *df* = 3; post hoc tests: A1-A2: *p* = 1.000, A1-A3: *p* = 0.257, A1-A4: *p* = 0.180, A2-A3: *p* = 0.034, A2-A4: *p* = 0.014, A3-A4: *p* = 0.317).

#### Reading and Monologue Intelligibility

**Fig. 1:**
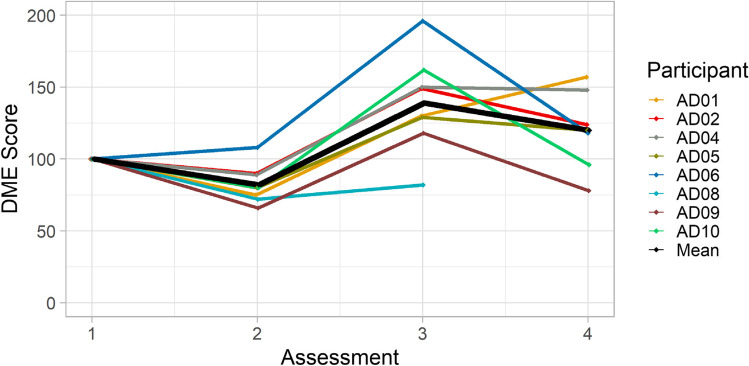
Reading Intelligibility across assessment session by speaker and group mean

The individual performance patterns (Fig. 1) as well as Friedman test results for the reading intelligibility data indicates significant changes across the four assessment sessions (Friedman test: *p* = 0.001, *χ*^2^ = 16.54, *df* = 3). Post hoc tests indicate this was due to significant differences across the two pre-treatment assessments (A1-A2: *p* = 0.017), as well as between pre- and post-treatment performance (A1-A3: *p* = 0.021, A2-A3: *p* = 0.012, A1-A4: *p* = 0.128, A2-A4: *p* = 0.018). Intelligibility changes post-treatment did not reach significance (A3-A4: *p* = 0.091). However, rather than reflecting a stabilising of intelligibility levels longer term, this result was due to a more variable performance across participants (Fig. 1). Whilst some remained relatively stable or even improved further between assessments 3 and 4, others dropped again in level. However, the majority of participants remained above pre-treatment levels, with exception of AD9 and AD10 who returned to their original levels in A4. It should also be noted that one participant (AD8) did not show any improvements post-treatment although his final assessment data are missing.

In summary, seven of the eight speakers analysed improved post-treatment, and for five of these this improvement was sustained longer term.

In contrast to the reading data, the monologue did not show significant changes between any of the assessment points (Friedman test: *p* = 0.125, *χ*^2^ = 5.75, *df* = 3) due to variable individual performance.

#### Utterance to Utterance Variability

There was no significant difference in UUV values across the assessments (Friedman test: *p* = 0.968, *χ*^2^ = 0.257, *df* = 3). Performance was highly variable with no trends identifiable. The UUV measure was therefore not suitable to track progress after treatment for the current data.

#### Communication Participation and Confidence

**Fig. 2:**
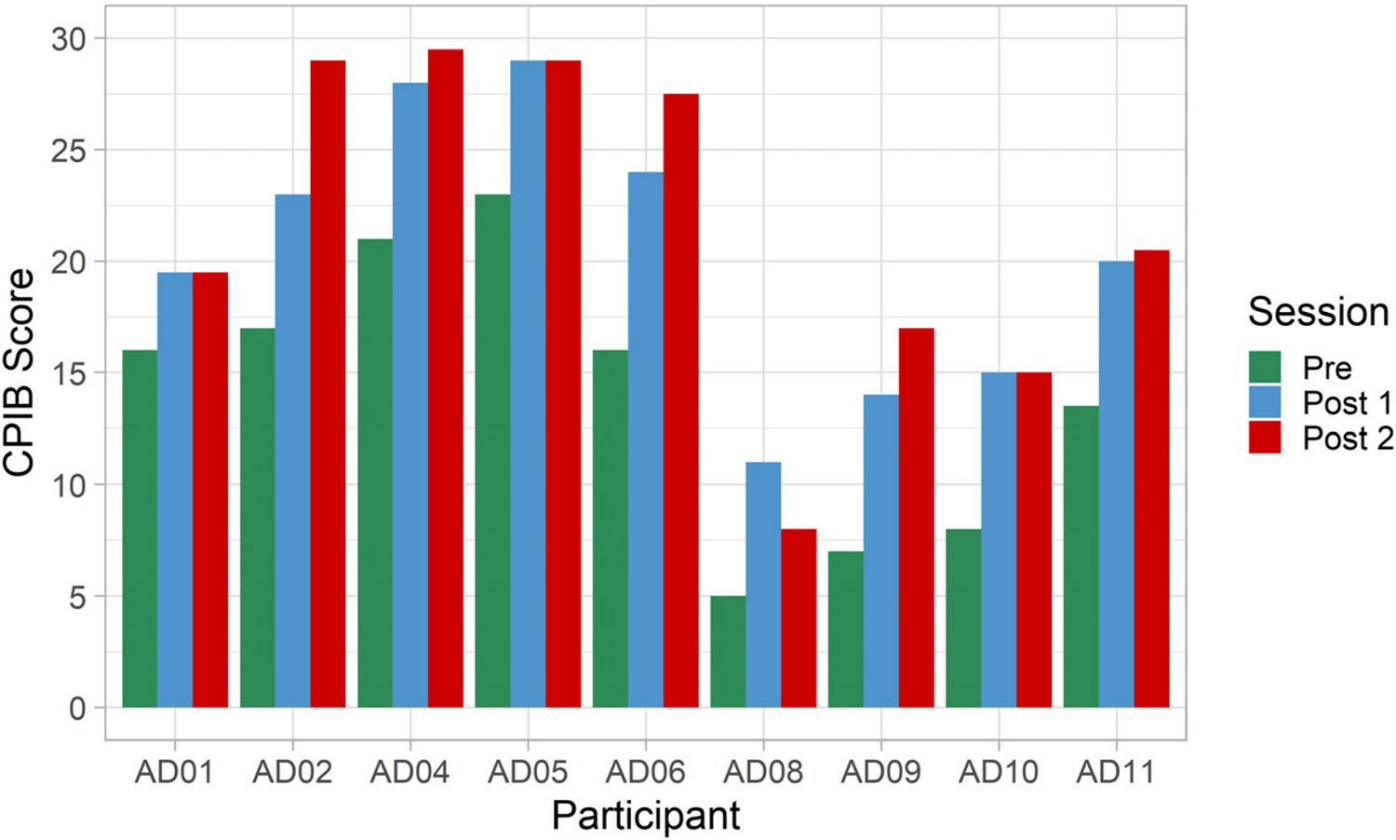
Communication Participation Item Bank (CPIB), maximum scores (no impact) = 30

The statistical analysis for communication participation (CPIB [54]) returned significant differences between pre- and post-treatment values and no difference between immediate and long-term post-treatment assessments (Friedman test: *p* < 0.001, *χ*^2^ = 15.70, *df* = 2; post hoc tests: A1-A3: *p* = 0.007, A1-A4: *p* = 0.008, A3-A4: *p* = 0.141). Individual data (Fig. 2) show that only one person (AD8) reported a reduction in scores from A3 to A4, which he attributed to reduced opportunities to communicate rather than his ataxia impacting more strongly on his communication again. In addition, both post-treatment scores are higher than his pre-treatment level, which is noteworthy in the absence of any intelligibility improvement in this participant. A similar picture is presented by the confidence ratings (Friedman test: *p* < 0.001, *χ*^2^ = 13.15, *df* = 2; post hoc tests: A1-A3: *p* = 0.011, A1-A4: *p* = 0.018, A3-A4: *p* = 0.414).

#### Participant Self-Perception of Their Communication

All participants reported positive outcomes of the intervention in A3 for both voice and articulation.

Several participants mentioned that their voice had become stronger, louder and more stable without sudden loudness bursts. One participant reported improvements in her intonation—“I don’t sound like a robot anymore” (AD11).

Participants also reported articulatory improvements such as speaking more clearly and deliberately, over-articulating and trying to pronounce every syllable. In addition, the “Clear” strategy had impacted participants’ pacing of speech, i.e. speaking slower, spacing words out and taking more breaths.

Furthermore, they reported the effort required to speak was lower after therapy, allowing participants to speak in longer utterances and for longer periods of time—“I’m now quite happily chatting away to the hairdresser for 30 min” (AD6). They also reported improved self-management of their speech efforts and increased motivation to persevere with conversation despite tiredness, which enables increased participation more, in particular at times or situations, such as in groups, when they would normally have withdrawn from communication.

#### Participant Feedback on the Therapy Process

Participants were positive about the intervention regime, indicating that it had addressed their needs and that they had a good understanding of the purpose and benefits of the strategies conveyed. They felt the balance between individual and group elements was good. The two more severely affected participants who required a further session in week 2 of the group phase indicated that this resolved their issues. Scheduling had not been a problem either and the online nature of the intervention was not seen as a barrier but rather as facilitating participation. Group dynamics had been good in both groups and members continued to meet socially on their own accord twice a week after the intervention ended to continue practicing together.

Participants also listed a range of social and speech benefits arising specifically from the group phase, as outlined in Table 3. Whilst the social benefits described could also have been achieved by attending a generic support group, the speech benefit were specifically related to the current therapy model. Two participants who had recently attended individual speech and language therapy indicated that the group meetings had added benefits for them. In addition, a further two participants commented that having to lead the groups themselves helped reinstate former social roles, e.g., they felt more in charge again.

**Table 3:** Themes related to group intervention benefits

Social benefits	Speech benefits
Meeting other people with ataxia	Opportunity to talk, everybody gets a turn
Feeling you’re not the only one who has the problem	Feedback from others / constructive criticism valuable to learning
Find out more about different ataxia presentations and severities and associated problems	Taking cues from one another / hearing others use speech strategies helps and motivates to integrate them into own speech
Sharing coping strategies	Helps conquer apprehension about speaking in a more supportive environment / gives confidence talking with others
Sharing frustrations	Improves motivation to practise
Psychological support	Allows practice of real-life speaking situations, re-establishment of roles

## Discussion

The aim of this study was to pilot the feasibility, acceptability and potential effectiveness of ClearSpeechTogether in a small group of people with progressive ataxia and mild to moderate dysarthria.

In terms of feasibility and acceptability, recruitment was highly successful with a large waiting list of participants remaining on study completion. Retention to the study was at 80% with reasons for dropping out of the study based on significant personal circumstances. Adherence was also good.

Qualitative data indicates that participants found the programme addressed their communication needs, and that the content, scheduling and delivery of treatment were appropriate. No adverse effects were reported from the treatment and it did not impact participants’ fatigue levels. Group dynamics were also positive. Our study thus concurs with previous reports of psychosocial benefits of group intervention (e.g., [43, 44]).

There were no access issues related to the online provision in our study, and all participants managed the technology without assistance. However, this aspect requires monitoring in future studies to ensure equity of access to all who require treatment.

In terms of acceptability to service providers, the current model demonstrated added value compared to previous research into group intervention by incorporating peer-led treatment sessions. This maximised an average clinician time commitment of 5–6 sessions per patient, comparable to standard NHS input, whilst providing a total of 24 sessions for each patient. Whilst some additional technical support was put in place via non-clinical volunteers, this could be phased out quickly and should require relatively little time commitment. Future research will need to formally investigate the health economic benefits and feasibility of the group approach within a clinical context.

In relation to communication outcome measures, the preliminary results indicate limited physiological improvements, though they show promising increases in reading intelligibility as well as communication participation and confidence.

The lack of improvement in relation to maximum phonation time was not surprising given that the majority of participants’ pre-treatment performance fell within the normal range [25, 26, 61]. Importantly, those with considerably reduced MPTs did show some improvements. In relation to voice quality, our participant sample generally had no or very mild impairment, similar to previous reports [12, 25, 27, 28]. In contrast to our earlier study [25], strain was the only aspect to improve significantly this time. This might be explained by a different baseline voice profile of the participants and the difference in aetiology.

The results for reading intelligibility were promising and in line with other therapies [24, 27, 29]. The results were less clear for the monologue task due to variable performance of participants. With hindsight, too much focus on reading and not enough free speech practice was built into the exercise programme. This will be adjusted in future trials. Irrespective of the monologue results, the current study provides preliminary evidence that the programme can be successful in improving intelligibility, thus warranting further investigation.

The intervention also had a positive impact on communication participation and confidence comparable to some other treatment investigations for ataxia [25, 27, 28, 31]. It is noteworthy that this could be achieved through virtual, online interactions without the necessity of face to face meetings. Without a comparator treatment these improvements cannot be directly attributed to the speech intervention and could simply be a function of participation in a programme, as suggested by AD8’s results, who showed no notable improvements in intelligibility but reported an increase in confidence and participation. Comments from participants whose psychosocial scores dropped longer term suggest that additional communication opportunities beyond the therapy group were important to maintain the gains made during treatment. This highlights the importance of monitoring activity outside clinic in future investigations into psycho-social benefits of interventions. The results also raise the question whether intelligibility remains the most appropriate core outcome measure for trials with patients with progressive dysarthria or whether this should be supplemented or superseded by measures that better reflect participants’ activity and participation in everyday communication and measures of change in the impact of their speech disorder.

Finally, the qualitative evaluations stressed the added benefits the group treatment provided for both speech and psychosocial factors, similar to previous research on group interventions (cf. Whillans et al. [32] for a review). In addition, we identified specific benefits of the peer led design as reflected in the reports of positive changes to social roles in terms of having to take charge of the session. Social role limitations were highlighted as the second most impactful problem in people with FRDA in a recent interview study [62]. These can be difficult to address directly in a one–one-therapeutic context, rendering our result particularly encouraging in this respect.

## Conclusion

Whilst the above reported outcomes are generally positive, it has to be stressed that they are based on a small number of results and experiences. This considerably limited the statistical power and introduced inconsistencies due to individual performance variations. Nevertheless, we were able to demonstrate post-therapy improvements in the most important outcome measures, intelligibility and communication participation and confidence, highlighting the potential of the approach.

In conclusion, this study adds to the growing evidence of the fact that speakers with progressive ataxia can benefit from speech and language therapy, and confirms earlier reports on the (added) value of group intervention. Our study has taken existing group intervention models further by successfully introducing a patient-led element to the therapeutic regime which not only freed up clinician time,but also provided some additional benefits to addressing intractable psycho-social issues in our small group of participants. The current study thus provides an encouraging basis for further research into speech treatment in speakers with progressive ataxia as well as other related speech disorders, and into new models of treatment delivery that reduce the workload pressures of clinicians whilst maximising for patients.
